# The Stiff Side of Cancer: How Matrix Mechanics Rewrites Non-Coding RNA Expression Programs

**DOI:** 10.3390/ncrna12010007

**Published:** 2026-02-18

**Authors:** Alma D. Campos-Parra, Jonathan Puente-Rivera, César López-Camarillo, Stephanie I. Nuñez-Olvera, Nereyda Hernández Nava, Gabriela Alvarado Macias, Macrina Beatriz Silva-Cázares

**Affiliations:** 1 Instituto de Salud Pública, Universidad Veracruzana (UV), Av. Dr. Luis Castelazo Ayala s/n, Col. Industrial Ánimas, Xalapa 91190, Mexico; 2 División de Investigación, Hospital Juárez de México, Mexico City 07760, Mexico; 3 Posgrado en Ciencias Genómicas, Universidad Autónoma de la Ciudad de Mexico, Mexico City 03100, Mexico; 4 Departamento de Biología Celular y Fisiología, Instituto de Investigaciones Biomédicas, Universidad Nacional Autónoma de México, Mexico City 04510, Mexico; 5 Unidad Académica Multidisciplinaria Región Altiplano, Universidad Autónoma de San Luis Potosí, Carretera a Cedral km 5 + 600 Ejido San José de las Trojes, Matehuala 78700, Mexico

**Keywords:** matrix stiffness, miRNAs, lncRNAs, mechanotransduction, cancer

## Abstract

Extracellular matrix (ECM) stiffening is a defining biophysical feature of solid tumors that reshape gene regulation through mechanotransduction. Increased collagen crosslinking and stromal remodeling enhance integrin engagement, focal-adhesion signaling and force transmission to the nucleus, where key hubs such as lysyl oxidase (LOX), focal adhesion kinase (FAK) and the Hippo co-activators YAP1 and TAZ (WWTR1) promote proliferation, invasion, stemness and therapy resistance. Here, we synthesize evidence that quantitative changes in matrix stiffness remodel the miRNome and lncRNome in both tumor and stromal compartments, including extracellular vesicle cargo that reprograms metastatic niches. To address heterogeneity in experimental support, we classify mechanosensitive ncRNAs into studies directly validated by stiffness manipulation (e.g., tunable hydrogels/AFM) versus indirect associations based on mechanosensitive signaling, and we summarize physiological versus pathophysiological stiffness ranges across tissues discussed. We further review competing endogenous RNA (ceRNA) networks converging on mechanotransduction nodes and ECM remodeling enzymes, and discuss translational opportunities and challenges, including targeting mechanosensitive ncRNAs, combining ncRNA modulation with anti-stiffening strategies, delivery barriers in dense tumors, and the potential of circulating/exosomal ncRNAs as biomarkers. Overall, integrating ECM mechanics with ncRNA regulatory circuits provides a framework to identify feed-forward loops sustaining aggressive phenotypes in rigid microenvironments and highlights priorities for validation in physiologically relevant models.

## 1. Introduction

Matrix stiffness is now recognized as a central hallmark of solid tumors, acting not only as a structural cue but as a dynamic regulator of gene expression through mechanotransduction [1,2]. Increased collagen crosslinking, fibrosis and stromal remodeling elevate the elastic modulus of tissues, promoting integrin engagement, focal adhesion assembly and force transmission to the nucleus via the cytoskeleton and the LINC (Linker of Nucleoskeleton and Cytoskeleton)–lamina complex [3,4]. This mechanical input converges on mechanosensitive hubs such as lysyl oxidase (LOX), focal adhesion kinase (FAK) and the Hippo co-activators YAP1 and TAZ, driving proliferation, invasion, stemness, therapy resistance and pre-metastatic niche formation [5].

In parallel, accumulating evidence indicates that non-coding RNAs (ncRNAs)—particularly microRNAs (miRNAs) [6], long non-coding RNAs (lncRNAs) [7] and circular RNAs (circRNAs) [8]—are both mechano-responsive and capable of tuning these signaling nodes through competing endogenous RNA (ceRNA) networks [9,10,11]. In this review, we summarize how defined changes in matrix stiffness remodel the miRNome and lncRNome. We highlight stiffness-regulated miRNAs (e.g., miR-1246, miR-17-5p, let-7d-5p, miR-365a-5p, miR-3682-3p) and lncRNAs (NEAT1, PVT1, lnc-ZNF136, SNHG9) that control pathways such as PI3K/Akt, glycolytic metabolism and angiogenesis, in both tumor and stromal compartments and in exosomal cargo. We then discuss ceRNA circuits in which ncRNAs converge on *LOX*, FAK, YAP1 and TAZ, including LINC01270–/HIF1A-AS2/miR-29c–LOX, KCNQ1OT1–miR-211-5p–Ezrin/FAK/Src, LINC01811–miR-214-3p–YAP1 and MIR4435-2HG–miR-125a-5p–TAZ. Although most of these studies do not manipulate stiffness directly, they reveal post-transcriptional layers that can amplify or dampen mechanotransduction outputs at key structural and nuclear nodes. Finally, we outline current gaps and future directions, emphasizing the need for mechano-controlled 3D models, organoids and co-culture systems to validate which ncRNA–ceRNA axes are truly mechanosensitive in vivo. We propose that integrating biophysical control of ECM stiffness with ncRNA-based interventions (antagomiRs, mimics, antisense oligonucleotides, LOX inhibitors) may offer novel combinatorial strategies to disrupt the feed-forward loop between rigid matrices, ncRNA networks and aggressive tumor phenotypes in solid cancers.

Beyond cytoplasmic actin remodeling at focal adhesions, actin can also polymerize in the nucleus (nuclear F-actin), where it contributes to chromatin organization and the DNA damage response. Nuclear actin assembly can promote the spatial clustering and mobility of DNA double-strand breaks to facilitate homology-directed repair, and altered nuclear actin dynamics have also been implicated in epithelial–mesenchymal transition (EMT) programs and invasive phenotypes [12,13].

### 1.1. Composition, Structure and Stiffness of the Extracellular Matrix

The extracellular matrix (ECM) is an essential component of connective tissues that forms a complex three-dimensional network of structural macromolecules such as collagens, glycoproteins, elastin and proteoglycans [14]. In addition to providing physical support and defining the spatial organization of cells, the ECM acts as a biochemical and biomechanical platform that regulates fundamental processes such as cell differentiation, proliferation and migration [15,16]. Cells interact with the ECM through specialized receptors, mainly integrins, which allow them to perceive the physicochemical characteristics of their environment and adjust their behavior through mechanosensing and biochemical feedback mechanisms, thereby ensuring tissue homeostasis and proper organ function [17]. The composition and architecture of the ECM determine the biomechanical properties of the tissue, among which stiffness and viscoelasticity are particularly relevant. Stiffness is defined as the ability of a tissue to resist elastic deformation and is quantified by the Young’s modulus, expressed in kilopascals (kPa).

This parameter, which measures the elasticity of the material, is obtained using techniques such as atomic force microscopy (AFM) or rheometry, and allows comparison of stiffness differences between tissue types or between physiological and pathological conditions [2,5,18]. These properties are dynamic and can be modified by the cells themselves by altering the density, crosslinking or degradation of matrix macromolecules [14]. Factors such as collagen content and crosslinking, LOX activity, non-enzymatic glycosylation and the activity of matrix metalloproteinases (MMPs) directly modulate the elasticity of the cellular environment [3,19,20,21]. Likewise, pore size and fibrillar organization influence the diffusion of molecules and cell migration [22,23].

### 1.2. ECM Stiffness and Remodeling During Oncogenesis

During oncogenesis, the ECM acquires a decisive role as a physical and biochemical regulator of the tumor microenvironment [24]. In cancerous tissues, fibroblasts—particularly cancer-associated fibroblasts (CAFs)—increase the production of collagen, fibronectin and hyaluronic acid (HA), promoting matrix accumulation and reorganization [25]. Type I collagen, the main structural component, becomes more abundant and its fibers undergo enhanced enzymatic crosslinking mediated by LOX, which increases tensile strength and tissue stiffness [3,26]. This stiffening, in turn, alters cell signaling and activates mechanosensitive pathways that promote tumor cell survival, proliferation and invasion [27]. Fibronectin, also secreted by fibroblasts, contributes to the structural reinforcement of the stroma through its insoluble form and exerts additional regulatory functions by interacting with integrins and growth factors, thereby promoting migration, invasion and collagen reorganization [28,29].

These structural changes do not occur in isolation: they are accompanied by a reduction in pore size, an increase in local viscosity and a loss of fibrillar order, all of which modify nutrient diffusion, cell migration and drug response [22,30,31]. In addition, the tumor ECM is subject to constant remodeling driven by the combined action of cytokines, growth factors and MMPs secreted by stromal and tumor cells [20,32]. This process leads to a disorganized and stiff microenvironment in which cancer cells interact with the matrix at every step of the metastatic cascade. Taken together, increased collagen density, crosslinking and stiffness, along with the overproduction of fibronectin and HA, create an abnormal physical setting that favors tumor progression. Thus, tumor microenvironment stiffness not only reflects structural changes in the matrix but also represents a key biomechanical determinant of cancer progression, capable of influencing signaling, migration and cell fate.

### 1.3. Cytoskeletal Architecture and Cell Adhesion Mechanisms

The cytoskeleton is a dynamic network of filaments that provides shape, organization and mechanical support to cells, while also coordinating their movement, division and structural stability [33]. It is mainly composed of actin microfilaments, microtubules and intermediate filaments, whose components cooperate to maintain cellular integrity and respond to environmental changes [33,34]. Among these, actin microfilaments play a central role in cell adhesion and migration [35]. Polymerized actin (F-actin) is organized into specialized structures—such as lamellipodia, filopodia and stress fibers—that regulate cell shape and motility [36]. Its dynamics depend on a balance between polymerization and depolymerization, controlled by proteins such as Arp2/3, formins, cofilin and Rho GTPases, which tune cytoskeletal architecture according to cellular demands [37,38]. The interaction between actin and myosin II, under the control of the RhoA–ROCK pathway, generates contraction and force, which are essential for migration and maintenance of internal tension [35,39]. Integrins, transmembrane receptors formed by α and β subunits, act as anchoring points between the cell and the ECM [17,40].

These proteins bind components such as collagen, fibronectin and laminin, and connect to the cytoskeleton through adaptor proteins—talin, vinculin and paxillin—which transmit mechanical stability and organize the cell interior [41,42]. Depending on the combination of subunits, integrins regulate processes such as adhesion, migration, proliferation and survival, and cooperate with other signaling pathways, including those mediated by receptor tyrosine kinases (RTKs) [17,40]. Upon integrin engagement with their ligands, focal adhesions are formed: transient multiprotein complexes that link the plasma membrane to actin filaments. They initially arise as nascent adhesions that recruit proteins such as FAK, talin and vinculin, stabilizing the interaction with the ECM [43]. As internal tension generated by the cytoskeleton increases, adhesions mature and organize into structures aligned with stress fibers, strengthening cell anchorage and controlling motility [44]. Altogether, the coordination between the cytoskeleton, integrins and focal adhesions constitutes a highly regulated system that supports cellular architecture and enables physical interaction with the environment [45]. This network not only provides structural support, but also defines the capacity of the cell to adhere, migrate and maintain its spatial organization within continuously remodeling tissues [46]. In this context, cytoskeletal architecture and cell–matrix interactions provide the physical basis upon which cells interpret their surroundings. The arrangement of actin filaments, integrin-mediated anchorage and the formation of focal adhesions not only ensure structural stability, but also create a system capable of sensing changes in tension, stiffness and composition of the ECM. Once organized, these structural elements set the stage for the processes through which cells can translate physical cues into coordinated molecular responses, giving rise to the mechanotransduction mechanisms that govern cellular adaptation to external and internal forces.

## 2. Cell Mechanotransduction: From Physical Forces to Biochemical Signals

**Figure 1:**
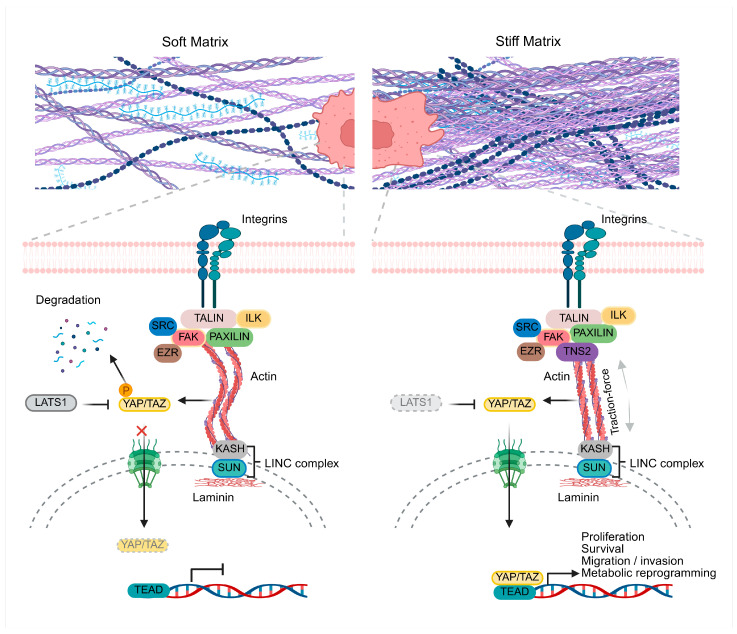
Extracellular matrix stiffness regulates YAP/TAZ-mediated mechanotransduction. In a soft matrix (**left**), the loosely organized fibers generate weak integrin-mediated adhesions, with unstable adhesion complexes (talin, paxillin, ILK, FAK, SRC, EZR) and actin filaments that favor their degradation. Under these conditions, LATS1 remains active and phosphorylates YAP/TAZ, promoting their cytoplasmic retention and preventing nuclear accumulation and TEAD activation. In a stiff matrix (**right**), the increased fiber density promotes the assembly of mature focal adhesions connected to actomyosin stress fibers that transmit traction forces. Integrin adaptors such as TNS2, which localize to focal and fibrillar adhesions, contribute to coupling integrins to the actin cytoskeleton and to efficient force transmission to the nucleus through the LINC complex (SUN–KASH) and the nuclear lamina. LATS1 inactivation allows YAP/TAZ dephosphorylation and nuclear translocation, where they associate with TEAD to induce transcriptional programs linked to proliferation, survival, migration/invasion, and metabolic reprogramming. Up and black arrows indicate activation and black inhibition arrow indicates suppression.

The ECM is an essential component of the tissue microenvironment, not only because it provides structural support and defines epithelial cell polarity, but also because it acts as a dynamic signaling platform [24]. Its molecular composition of collagen, fibronectin, laminin and proteoglycans allows the storage and release of biochemical cues that regulate adhesion, migration, differentiation and survival [47]. However, beyond its chemical composition, the mechanical properties of the ECM such as stiffness, tension and elasticity are key determinants of cellular activity. Mechanotransduction is the process by which cells sense these physical signals and convert them into specific biochemical responses [2,5]. This mechanism begins with the detection of changes in stiffness or tension through mechanosensitive receptors, such as integrins, Piezo1 ion channels and cadherins [45,48,49]. Upon binding to matrix proteins such as fibronectin or collagen, integrins adopt an active conformation and recruit adaptor proteins (talin, vinculin, paxillin) and kinases such as FAK, Src and ROCK, which assemble into focal adhesions [39,50]. These structures connect the ECM to the actin cytoskeleton, allowing mechanical forces to propagate to the nucleus through the LINC complex and nuclear lamins, thereby altering chromatin organization and activating mechanosensitive transcription factors such as YAP/TAZ or NF-κB [51,52]. Thus, cells translate physical stimuli into molecular responses that modulate their shape, proliferation and fate, establishing a feedback circuit that can either maintain homeostasis or, under pathological conditions such as cancer, reprogram gene expression toward more aggressive and therapy-resistant phenotypes [2,27]. Notably, recent mechanobiology studies have shown that microtubule architecture regulates YAP/TAZ mechanotransduction through AMOT stability, while mechanosensitive ion channels such as Piezo1 activate YAP/TAZ to promote ECM remodeling, reinforcing YAP/TAZ as central hubs linking mechanical forces to transcriptional and matrix-remodeling programs [53,54] (Figure 1).

Experimentally, increased tissue stiffness has been shown to act as an early risk factor in various cancer types. In breast cancer, tissues with high mammographic density display a stiffer stroma, characterized by greater collagen alignment and epithelial proliferation, features associated with increased tumor risk [55,56]. Similarly, in prostate cancer, higher values of the Young’s modulus correlate with a greater risk of malignancy, suggesting that tissue stiffness could function as a mechanical predictor of neoplastic transformation [57]. In cell-based models, this relationship is also evident. For example, in non-tumoral mammary epithelium (MCF10A), a controlled increase in substrate stiffness significantly enhances cell proliferation and leads to the loss of differentiated acinar structures, directly linking the mechanics of the microenvironment with cell cycle control [54]. In three-dimensional PEGDA hydrogels, an intermediate stiffness of about 5 kPa provides an optimal niche for breast cancer cells, selectively expanding subpopulations with cancer stem cell (CSC)-like properties, as evidenced by robust tumorsphere formation, enrichment of CD44^+^/CD24^−^ cells, and upregulation of CSC and EMT markers together with increased YAP/TAZ activity. Notably, this optimum stiffness shifts to ~25 kPa for colorectal and gastric cancer cells and to ~50 kPa for osteosarcoma, indicating that CSC maintenance is tuned to tissue-of-origin-specific mechanical cues [58].

In prostate cancer, ECM stiffening has been shown to increase resistance to docetaxel, reducing drug-induced apoptosis and altering the expression of genes associated with mechanotransduction [59]. Finally, this mechanical dialog is bidirectional: cells not only respond to the physical properties of their environment, but also readjust their own mechanical state through cytoskeletal remodeling and the secretion of matrix-modifying enzymes such as LOX or MMPs, thereby altering ECM stiffness. This phenomenon, known as dynamic reciprocity, creates a continuous feedback loop that maintains tissue homeostasis under normal conditions but, in cancer, perpetuates a stiff, inflammatory and pro-tumor microenvironment that favors progression and therapeutic resistance [60,61]. In this setting, the role of ncRNAs as fine mediators of mechanotransduction has gained particular relevance [62]. Beyond classical transcription factors and changes in protein-coding genes, matrix stiffness and the traction forces transmitted to the nucleus reconfigure the expression landscape of miRNAs and lncRNAs, modulating the stability and translation of hundreds of transcripts involved in adhesion, ECM remodeling, cell cycle control and cell death [62,63]. In turn, these non-coding transcripts reciprocally regulate the expression of integrins, cytoskeletal components, focal adhesion kinases and ECM-remodeling enzymes, establishing feedback loops that amplify or attenuate mechanical signals. Thus, they not only act as sensors and effectors of matrix stiffness but also help lock in aggressive and drug-resistant phenotypes in cancer, opening the possibility of using them as mechano-dependent biomarkers and as therapeutic targets.

Table 1 summarizes representative tissue stiffness discussed in this review and underscores how baseline tissue mechanics may shape ncRNA mechanosensitivity (values depend on measurement modality).

### 2.1. Mechanosensitive MicroRNAs and Extracellular Matrix Stiffness

MicroRNAs (miRNAs) are small ncRNAs of ~18–25 nucleotides that regulate gene expression post-transcriptionally by binding to complementary sequences in target mRNAs, thereby promoting their degradation or inhibiting their translation [67]. This fine-tuned control of gene networks is essential for processes such as proliferation, differentiation, apoptosis, migration and tissue remodeling [68]. In cancer, miRNAs can act as oncogenes or tumor suppressors, and their dysregulation contributes to tumor progression, metastasis and therapeutic resistance [69]. Increasing evidence indicates that mechanical signals from the microenvironment, including extracellular matrix stiffness, modulate the cellular and vesicular miRNome, generating mechanosensitive miRNA signatures that link mechanotransduction to the reprogramming of gene expression. In a three-dimensional model of human oral squamous cell carcinoma, Cal27 cells cultured in hydrogels characterized as “stiff” (0.9 kPa) showed that matrix stiffness profoundly remodels the miRNome associated with tumor progression.

Differential expression analysis identified miRNAs whose abundance was significantly modulated by stiffness, with a set of upregulated oncogenic DEMs that included miR-1246, previously reported to promote motility and invasion in OSCC; miR-30a-3p, which activates Akt signaling to drive metastasis; as well as miR-455-5p and miR-574-5p, both proposed as prognostic biomarkers in different tumor types. In contrast, stiffness repressed miRNAs with tumor-suppressive functions—such as miR-200c-3p and miR-125a-5p—whose downregulation has been associated with metastatic disease and with small extracellular vesicle derived from head and neck squamous cell carcinoma cell lines, respectively. Functional analysis of these DEMs revealed enrichment in pathways with pro-tumoral associations, including the MAPK cascade, epithelial cell proliferation, and the regulation of cell migration and motility, suggesting that matrix stiffness acts not only as a mechanical stimulus but also as a key regulator of miRNA networks that favor invasive and metastatic phenotypes [70].

The influence of stiffness on the miRNome has also been documented in hepatocellular carcinoma (HCC). In MHCC97H and Hep3B cells cultured on gels with stiffness values of 6 kPa (normal liver) versus 16 kPa (cirrhosis), to mimic distinct hepatic microenvironments, increased stiffness was found to activate the expression of miR-17-5p. This miRNA reduces *PTEN* expression and consequently activates the PI3K/Akt pathway, thereby promoting cell invasion. These findings directly link hepatic microenvironment stiffness to the dysregulation of oncogenic miRNAs and highlight the miR-17-5p/PTEN/PI3K–Akt axis as a mechanosensitive module that drives HCC progression [71]. In addition to reprogramming the intracellular miRNome, extracellular matrix stiffness also modulates the miRNA content packaged into extracellular vesicles and their impact on distant metastatic sites. In a model examining the formation of pre-metastatic niches in the lung, human MHCC97H and Hep3B cells, together with the murine Hepa1-6 line, were cultured on gels of 6 kPa (normal tissue) and 16 kPa (cirrhosis).

**Figure 2:**
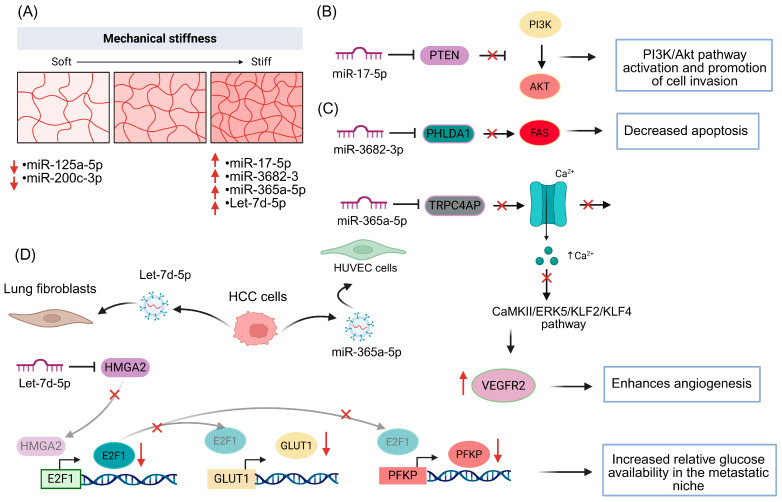
Mechanical stiffness-regulated miRNAs and their effects on tumor progression. (**A**) Increasing ECM stiffness upregulates miR-17-5p, miR-3682-3p, miR-365a-5p and let-7d-5p, whereas miR-125a-5p and miR-200c-3p decrease in soft matrices. (**B**) miR-17-5p represses PTEN, leading to PI3K/Akt pathway activation and enhanced cell invasion. (**C**) miR-3682-3p targets PHLDA1 and reduces FAS signaling, decreasing apoptosis. (**D**) HCC-derived exosomal let-7d-5p is taken up by lung fibroblasts, repressing HMGA2, lowering *E2F1*, *SLC2A1* (*GLUT1*) and *PFKP* transcription, which reduces glucose uptake and increases glucose availability in the metastatic niche. In parallel, miR-365a-5p inhibits *TRPC4AP*, reducing Ca^2+^ influx and inactivating the CaMKII/ERK5/KLF2/KLF4 pathway, ultimately increasing VEGFR2 expression and angiogenesis. Up red arrows and black arrows indicate activation, down red arrows, black inhibition arrows and red cross indicate suppression.

The stiffer condition increased the release of exosomes enriched in let-7d-5p and miR-365a-5p, which reprogram the pulmonary microenvironment. Exosomal let-7d-5p derived from HCC represses *HMGA2* in lung fibroblasts, altering *E2F1* acetylation and reducing the expression of SLC2A (GLUT1), PFKP and HK2, thereby decreasing glucose uptake by fibroblasts and promoting glycogen accumulation, which results in a relative enrichment of glucose in the metastatic niche [72]. In parallel, miR-365a-5p is packaged into HCC exosomes and taken up by endothelial cells (HUVECs), where it represses TRPC4AP, reducing Ca^2+^ influx and inactivating the CaMKII/ERK5/KLF2/KLF4 pathway [72]. This leads to decreased ZO-1 and VE-cadherin, but increased VEGFR2, thereby enhancing angiogenesis and vascular permeability. Together, these findings link primary tumor stiffness to metabolic and vascular reprogramming in the lung and highlight stiffness-sensitive exosomal miRNAs as key mediators in the establishment of the pre-metastatic niche [72]. Another relevant example is miR-3682-3p, whose expression increases under conditions of high matrix stiffness (25.6 kPa) in MHCC97H cells cultured on polyacrylamide hydrogels. Inhibition of this miRNA reduces proliferation, induces apoptosis and limits tumor growth in murine models [73]. Luciferase assays demonstrated that PHLDA1 is a direct target of miR-3682-3p; its repression attenuates activation of the FAS receptor, thereby altering apoptotic and autophagic processes. These findings suggest that tumor microenvironment stiffness promotes proliferation and suppresses programmed cell death in HCC through the miR-3682-3p/PHLDA1/FAS axis, and reinforce the notion that miRNAs act as molecular transducers of mechanical signals into survival and growth decisions [73](Figure 2).

Taken together, these studies show that extracellular matrix stiffness reconfigures miRNA expression patterns both in tumor cells and in the extracellular vesicles they release, converging on signaling pathways such as PI3K/Akt, MAPK, glycolytic metabolism, cytoskeletal dynamics and angiogenesis. From this perspective, mechanosensitive miRNAs emerge as central nodes that integrate the physicochemical signals of the microenvironment with tumor phenotypic plasticity, and they are increasingly viewed as potential biomarkers and therapeutic targets in settings of elevated ECM stiffness. Table 1 summarizes direct (e.g., tunable hydrogels/PDMS) or indirectly associated via mechanosensitive signaling without stiffness manipulation.

Future studies should build upon mechanobiology-oriented tools to strengthen causal inference and translational relevance. First, it is important to distinguish ncRNAs, whose mechanosensitivity has been validated under controlled stiffness conditions (e.g., tunable hydrogels with defined kPa ranges and independent mechanical verification), from ncRNAs that are linked indirectly through downstream markers of mechanosignaling. Second, because baseline stiffness differs markedly across organs and disease stages, mechanosensitive thresholds should be interpreted in a tissue-contextual manner; representative physiological and pathophysiological stiffness ranges for multiple tissues and cancers are summarized in Table 2.

### 2.2. Mechanosensitive lncRNAs and Extracellular Matrix Stiffness

lncRNAs are transcripts longer than 200 nucleotides that, although they do not encode proteins, regulate gene expression at multiple levels: from chromatin remodeling and modulation of transcriptional complexes to the control of mRNA stability and translation, as well as the organization of subnuclear domains [74]. In cancer, numerous lncRNAs have been associated with proliferation, invasion, cancer stem cell plasticity, stress responses and drug resistance [75]. Analogous to miRNAs, lncRNAs are now recognized not only as responders to classical biochemical cues but also to mechanical signals derived from extracellular matrix stiffness, acting as key nodes that translate physical changes in the microenvironment into oncogenic or therapy-resistant transcriptional programs. A paradigmatic example is NEAT1, an essential lncRNA for the biogenesis of nuclear paraspeckles. In cancer cells cultured on soft (3 kPa) versus stiffer (40 kPa) polyacrylamide hydrogels, soft substrates were associated with a higher number, area and size of paraspeckles, whereas stiff matrices suppressed paraspeckle formation. This suppression was strictly dependent on NEAT1, since its knockdown by RNA interference markedly reduced paraspeckles regardless of substrate stiffness. Traction-force-dependent mechanotransduction further contributed to this effect: non-muscle myosin II inhibition with blebbistatin on stiff matrices restored paraspeckle number and size to levels comparable to soft substrates, indicating that actomyosin contractility suppresses paraspeckle assembly. Together, these findings provide direct evidence that NEAT1-seeded paraspeckles mediate a nuclear mechano-response, integrating mechanical cues from the ECM and cytoskeleton into changes in nuclear organization and chromatin architecture [76].

In line with this, other studies have shown that matrix stiffness regulates lncRNA expression in ovarian cancer. In SKOV3, A2780 and HO8910 cell lines cultured on polydimethylsiloxane hydrogels with stiffness values of 106 kPa (stiff matrix) and 25 kPa (moderately stiff matrix), NEAT1 and LINC00894 were found to be overexpressed under stiffer conditions [7]. In contrast, the lncRNA SNHG8 showed reduced expression on stiff matrices. Functionally, SNHG8 inhibition by RNA interference decreased the efficiency of homologous recombination repair and increased sensitivity to etoposide and cisplatin, linking this lncRNA to DNA damage responses and chemoresistance [7].

Clinical analyses further revealed that SNHG8 expression correlates negatively with overall survival, suggesting that high levels of this lncRNA are associated with poorer prognosis and that its modulation could alter the response to chemotherapy within a mechanically altered ovarian cancer microenvironment [7]. Another mechanosensitive lncRNA of interest in ovarian cancer is PVT1. This lncRNA is upregulated when cells lose cell–cell contacts and when ECM stiffness increases, through a control axis dependent on YAP1, a classical mechanotransduction coactivator [77]. In SK-OV-3 cells cultured on polyacrylamide hydrogels with an elastic modulus of 8 kPa, stiffness-induced YAP1 activation elevates PVT1 expression, positioning it as a downstream effector of mechanosensitive signaling [77]. PVT1 knockdown reduces tumor dissemination and broadly impacts stress-response and drug-metabolism pathways (including the response to doxorubicin), thereby altering chemosensitivity. These data directly link matrix stiffness to lncRNA-dependent reprogramming of tumor progression and chemoresistance in ovarian cancer [77].

Matrix stiffness also reprograms the stromal compartment through lncRNAs. In human HFL1 lung CAFs cultured on collagen hydrogels of 0.2 kPa (soft condition) and 50 kPa (stiff condition), the expression of satellite lncRNAs HS2/HS3 was significantly induced under stiff conditions [78]. This induction was further enhanced by profibrotic and proinflammatory stimuli such as TGFβ1, IL1α or bleomycin. Deletion of satellite lncRNAs in CAFs reduced cellular senescence and blocked the establishment of an inflammatory senescence-associated secretory phenotype (SASP). At the transcriptional level, silencing HS2/HS3 markedly downregulated key inflammatory genes, including IL6, IL8, IL1B, and CXCL1, which are central mediators of the iCAF (inflammatory CAF) phenotype. Other myofibroblast-associated genes (such as COL1A1, COL4A1, ACTA2/αSMA, FN1, HAS2, and COL11A1) were not substantially affected, indicating that satellite lncRNAs primarily control the inflammatory transcriptional program rather than the myofibroblastic program [78]. Taken together, these findings identify satellite lncRNAs HS2/HS3 as mechano-dependent regulators of inflammation in the pulmonary tumor microenvironment and as potential therapeutic targets to modulate the interplay between stiffness, senescence, and inflammation.

lncRNA-dependent responses to stiffness also affect focal adhesion components and cytoskeletal organization. In SKOV-3 epithelial ovarian cancer cells cultured on polyacrylamide hydrogels with stiffness values of 1 kPa (soft), 6 kPa (normal) and 60 kPa (stiff), PLEC and TNS2, two genes localized at focal adhesions, were identified as being regulated by the lncRNA lnc-ZNF136, based on co-expression and cis/trans prediction analyses [11]. These data suggest that lnc-ZNF136 acts as a modulator of the cellular response to substrate stiffness, likely coordinating the expression of adhesion and anchoring genes that determine how EOC cells sense and adapt to a stiffer environment [11]. Complementing this substrate-dependent regulation, intrinsic modulation of cellular mechanical properties by lncRNAs has also been reported. An experimental study in lung carcinoma demonstrated that the lncRNA LINC00472 not only regulates cell migration and invasion but also increases cellular stiffness and adhesion by promoting cytoskeletal reorganization. In A549 and PC-9 cells, enforced expression of LINC00472 led to changes in actin and vimentin organization and strengthened cell–cell adhesion. Using AFM to assess mechanical properties, the authors showed that LINC00472 overexpression increased cellular stiffness and enhanced F-actin density and integrity, potentially through its interaction with YBX1. In parallel, LINC00472 upregulated E-cadherin expression, further reinforcing cell adhesion, and these coordinated biomechanical changes were associated with reduced migratory and invasive capacities [79].

**Figure 3:**
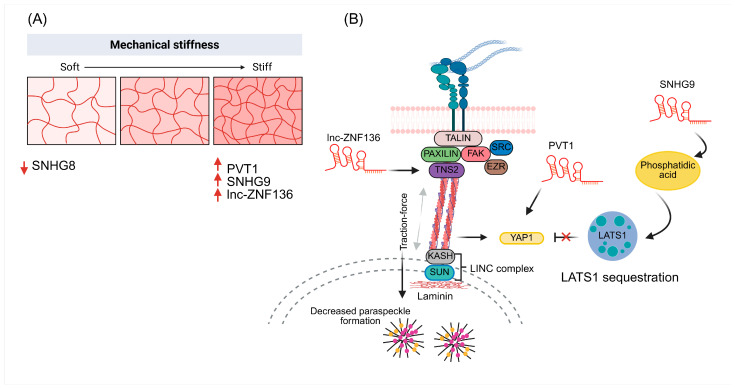
Stiffness-regulated lncRNAs integrate extracellular matrix mechanics with focal adhesion signaling, YAP1 activity and nuclear organization. (**A**) Schematic representation of a gradient of extracellular matrix stiffness from soft to stiff substrates. Increasing stiffness is associated with decreased expression of SNHG8 and upregulation of the stiffness-responsive lncRNAs PVT1, SNHG9 and lnc-ZNF136. (**B**) Proposed model of how these lncRNAs participate in mechanotransduction. In stiff matrices, lnc-ZNF136 associates with TNS2 and enhances its expression, thereby increasing the levels of TNS2 at integrin-based adhesion complexes that link to actin bundles and contribute to traction-force transmission to the nucleus through the LINC (SUN–KASH) complex and the nuclear lamina, leading to decreased paraspeckle formation. In parallel, PVT1 supports YAP1 activation, whereas SNHG9 promotes phosphatidic acid–dependent sequestration of LATS1 in cytoplasmic condensates, limiting LATS1-mediated inhibition of YAP1 and favoring its nuclear activity. Up red arrows and black arrows indicate activation, down red arrows and black inhibition arrows indicate suppression.

Finally, some lncRNAs act on central mechanotransduction nodes rather than responding directly to stiffness, yet are essential to amplify the oncogenic output of these pathways. In breast cancer, the lncRNA SNHG9 has been described as a modulator of YAP signaling in MDA-MB-231 cells. SNHG9 binds phosphatidic acid and promotes the formation of LATS1 liquid condensates through liquid–liquid phase separation (LLPS) [80]. In this context, LATS1 is sequestered into inactive droplets, which attenuates its kinase activity toward YAP and reduces YAP phosphorylation. As a result, YAP accumulates in the nucleus and its transcriptional activity is enhanced; this amplifies oncogenic signaling related to proliferation, survival and migration [80]. Taken together, these studies indicate that extracellular matrix stiffness not only reprograms lncRNA expression in tumor and stromal cells, but also intersects with mechanotransduction pathways such as YAP/TAZ, focal adhesion organization, DNA repair, senescence and drug response. From this perspective, mechanosensitive lncRNAs—including NEAT1, LINC00894, SNHG8, PVT1, HS2/HS3, lnc-ZNF136 and SNHG9—emerge as integrators of physical microenvironmental cues with pro-tumoral transcriptional programs, and stand out as attractive candidates for biomarkers and therapeutic targets in tumors developing within rigid microenvironments (Figure 3).

Notably, mechanosensitive ncRNAs are not confined to tumor cells. In the stromal compartment, matrix rigidity promotes fibroblast activation and the emergence of inflammatory CAF states, with distinct lncRNA programs (e.g., HS2/HS3 in iCAFs) that can reinforce cytokine signaling, ECM remodeling, and paracrine feedback to tumor cells. Mechanical cues also modulate endothelial and immune-cell phenotypes, and extracellular vesicles may transmit stiffness-adapted ncRNA signatures across compartments, suggesting that cell-type-specific mechanosensitive ncRNA networks (including ceRNA relationships) should be interpreted within the tumor–stroma context (Table 3) [78,80,81,82].

### 2.3. Regulation of Mechanosensitive Hubs by Competing Endogenous RNA (ceRNA) Networks

In addition to directly modulating the expression of stiffness-sensitive miRNAs and lncRNAs, mechanical cues from the microenvironment converge on a set of mechanosensitive hubs that integrate and amplify mechanotransduction, in part through competing endogenous RNA (ceRNA) networks. In these networks, lncRNAs, circRNAs, mRNAs and pseudogene-derived transcripts that share miRNA response elements “compete” for the binding of common miRNAs, acting as molecular sponges that redistribute miRNA activity across their targets [84]. This ceRNA-based layer of regulation can buffer or amplify stiffness-induced changes in miRNA levels, thereby fine-tuning the expression of adhesion molecules, cytoskeletal regulators and ECM-remodeling enzymes and stabilizing mechanically adapted, pro-tumor phenotypes. Among these, LOX, FAK, YAP1 and TAZ stand out, acting at different levels along the ECM–focal adhesion–nucleus axis [19]. LOX catalyzes collagen and elastin crosslinking, increasing extracellular matrix stiffness and organization, thereby promoting the formation of migration tracks, tumor invasion and pro-metastatic microenvironments [85]. FAK, located at focal adhesions, converts forces generated by integrins and the actin cytoskeleton into biochemical signals that control migration, survival and epithelial–mesenchymal transition [86,87]. In the nucleus, the co-activators YAP1 and TAZ, regulated by the Hippo pathway and by tensions transmitted through the LINC complex and the nuclear lamina, orchestrate transcriptional programs associated with proliferation, stemness, drug resistance and adaptation to stiff matrices [88,89,90].

Although many ceRNA network studies do not explicitly manipulate ECM stiffness, the identification of ncRNA → miRNA → LOX/FAK/YAP1/TAZ axes reveals an additional post-transcriptional layer of control over these mechanosensitive hubs and suggests that ncRNAs can finely tune the cellular response to matrix stiffness-derived signals.

In this context, LOX represents a key intersection between ECM remodeling and ceRNA networks. In studies on gastric tumors and adjacent tissue, as well as in the KATO III cell line, increased expression of LINC01270 and LOX was observed, accompanied by decreased levels of miR-29c-3p [9]. This expression pattern suggested the existence of a ceRNA axis in which LINC01270 acts as a sponge for miR-29c-3p, thereby de-repressing LOX expression. Functionally, LINC01270 silencing by siRNA in KATO III cells increased miR-29c-3p levels, reduced LOX expression and significantly decreased cell viability, supporting the role of this lncRNA as a key regulator of LOX-mediated tumor progression [9]. Complementarily, combined analyses of TCGA, GTEx and multiple GEO cohorts have shown that LOX is markedly overexpressed in gastric cancer and consistently associates with worse overall survival, progression-free survival and post-progression survival [10]. Using a systems biology approach based on the ceRNA hypothesis, an axis HIF1A-AS2–miR-29c–LOX was identified, in which the lncRNAs HIF1A-AS2 act as sponge for miR-29c, thereby promoting LOX overexpression [10]. Tumors with high LOX levels display immune infiltration signatures compatible with M2 macrophage polarization, a more immunosuppressive microenvironment and increased resistance to chemotherapeutic agents, positioning LOX as a node at the intersection of ECM stiffness, immune microenvironment remodeling and chemoresistance in gastric cancer [10].

Importantly, only a subset of studies directly quantifies ECM stiffness (e.g., atomic force microscopy (AFM) indentation or bulk rheology) after ncRNA perturbation. In many reports, the contribution of ncRNAs to a “stiffer” microenvironment is inferred from ECM-remodeling outputs such as collagen deposition and crosslinking, LOX/MMP activity, CAF activation, and mechanosensitive transcriptional signatures. Where direct mechanical measurements are unavailable, we interpret these ncRNAs as regulators of ECM-remodeling programs that are expected to shift tissue mechanics, and we highlight the need for future work combining ncRNA gain/loss-of-function with direct biophysical measurements in 3D matrices and tumor–stroma co-culture systems.

A similar pattern is observed around FAK, a classical integrator of signals derived from cell–matrix interactions. In bladder cancer, the circRNA circPICALM illustrates how ncRNAs can directly modulate this mechanotransduction node. In patient cohort analyses, circPICALM was found to be markedly decreased in tumor tissue compared with normal urothelium, and low levels were associated with advanced stage, high histological grade, lymph node involvement and poorer overall survival [8]. Mechanistically, circPICALM is predominantly localized in the cytoplasm and acts as a sponge for miR-1265, a microRNA with pro-invasive effects. Using RNA pull-down assays, luciferase reporter constructs and capture with biotinylated miRNAs, it was demonstrated that circPICALM sequesters miR-1265 and neutralizes its ability to repress STEAP4, a protein with anti-metastatic functions whose expression is reduced in bladder cancer [8]. STEAP4, in turn, physically interacts with FAK and blocks its autophosphorylation at tyrosine 397, a critical site for FAK activation at focal adhesions. When circPICALM is low and miR-1265 is high, STEAP4 levels decrease, FAK phosphorylation at Y397 increases, and an epithelial–mesenchymal transition program is favored [8]. Convergently, in tongue squamous cell carcinoma, the lncRNA KCNQ1OT1 has been characterized as a central regulator of proliferation and cisplatin resistance through a ceRNA axis converging on Ezrin/FAK/Src signaling [91]. From lncRNA expression analyses in tissues from cisplatin-sensitive versus cisplatin-resistant TSCC patients, KCNQ1OT1 emerged as the most overexpressed transcript in resistant samples. KCNQ1OT1 binds miR-211-5p, whose expression is reduced in resistant cells; depletion of KCNQ1OT1 increases miR-211-5p levels, whereas overexpression of this miRNA alone suppresses proliferation and restores cisplatin sensitivity, acting as a tumor-suppressive microRNA [91]. Luciferase assays identified Ezrin (EZR) as a direct target of miR-211-5p, and modulation of this axis impacts FAK and Src activation, directly linking the ncRNA network to a signaling node associated with focal adhesions and the cellular response to cell–matrix interactions [91].

At the nuclear level, several ceRNA axes converge on YAP1, a Hippo co-activator that is particularly relevant in contexts of elevated matrix stiffness. In colorectal cancer, the lncRNA LINC01811 has been identified as a promoter of tumor cell migration and invasion through a ceRNA axis converging on YAP1 [92]. In addition, mechanobiology studies that directly modulate substrate stiffness show that stiffer matrices promote colon cancer cell motility and invasion in a YAP1-dependent manner [93]. Prediction analyses and luciferase reporter assays demonstrated that LINC01811 acts as a sponge for miR-214-3p, a microRNA with tumor-suppressive functions in CRC. LINC01811 depletion increased miR-214-3p levels in HT29 cells, while this miRNA was found to be reduced in tumor tissues [92]. YAP1 was subsequently identified as a direct target of miR-214-3p, and it was shown that miR-214-3p overexpression decreases YAP1 levels, whereas miR-214-3p inhibition or YAP1 overexpression rescues the migration, invasion and EMT pattern lost upon LINC01811 silencing. Taken together, these data position the LINC01811/miR-214-3p/YAP1 axis as an oncogenic circuit that enhances the invasive capacity of colorectal cancer and exemplifies how lncRNAs can modulate YAP1 activity—a key node in matrix stiffness-sensitive pathways—by adding a ncRNA layer of regulation over mechanotransduction programs [92]. Analogously, TAZ (WWTR1), the paralog of YAP1, is also under the control of multiple ceRNA networks. In triple-negative breast cancer (TNBC), the circRNA hsa_circ_0091074 has been described as a modulator of TAZ [94]. In TNBC samples and breast cancer cell lines, hsa_circ_0091074 is overexpressed, whereas miR-1297 is downregulated, showing an inverse correlation that led to the proposal that this circRNA acts as a sponge for miR-1297. Functional studies demonstrated that increasing miR-1297 significantly reduces proliferation, colony formation, migration and invasion of breast cancer cells, accompanied by decreased TAZ protein levels [94].

**Figure 4:**
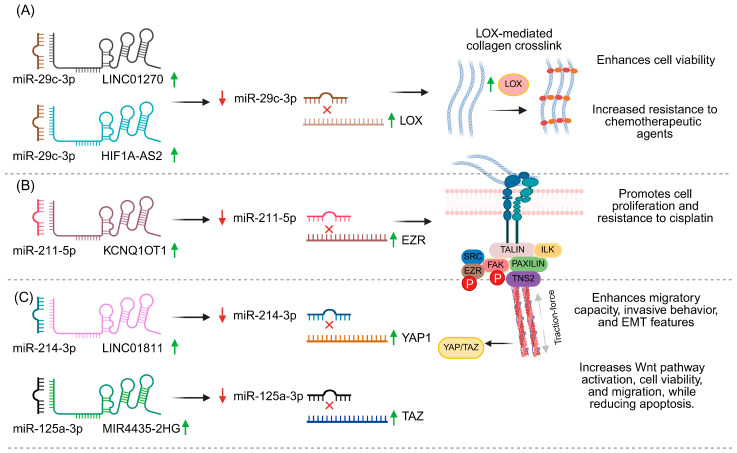
lncRNA–miRNA-mRNA regulatory axes that converge on ECM remodeling, focal adhesion signaling, and YAP/TAZ activity. (**A**) Upregulation of the lncRNAs LINC01270 and HIF1A-AS2 sequesters miR-29c-3p, leading to reduced availability of miR-29c-3p and increased LOX expression. Higher LOX activity enhances collagen crosslinking, which is associated with increased matrix stiffness, enhanced cell viability, and greater resistance to chemotherapeutic agents. (**B**) Increased expression of KCNQ1OT1 sponges miR-211-5p, resulting in decreased miR-211-5p levels and upregulation of EZR. (**C**) The lncRNAs LINC01811 and MIR4435-2HG act as competing endogenous RNAs for miR-214-3p and miR-125a-3p, respectively, decreasing these miRNAs and increasing YAP1 and TAZ expression. Activation of YAP/TAZ-dependent transcriptional programs enhances migratory and invasive capacities, EMT-related features, Wnt pathway activation, cell viability and migration, while reducing apoptosis. Up green arrows and black arrows indicate activation, down red arrows and red inhibition arrow indicates suppression.

Luciferase reporter assays confirmed that miR-1297 directly binds to the 3′UTR of TAZ and represses its expression, establishing miR-1297 as a suppressor of TAZ signaling [94]. Overall, these findings define a hsa_circ_0091074/miR-1297/TAZ circuit that drives TNBC aggressiveness and illustrate how circRNAs can potentiate the activity of mechanosensitive coactivators such as TAZ, adding a ncRNA layer of regulation to cellular responses triggered by extracellular matrix-derived signals. Finally, in neuroglioma, the lncRNA MIR4435-2HG represents another ceRNA example converging on TAZ. This lncRNA is overexpressed in glioma tissue compared with adjacent non-tumor tissue, whereas miR-125a-5p is downregulated and TAZ is upregulated [83]. In glioma cell models (U87 MG and U251), MIR4435-2HG is predominantly cytoplasmic and functions as a sponge for miR-125a-5p: luciferase reporter assays show that miR-125a-5p binds both MIR4435-2HG and the 3′UTR of TAZ, and that mutation of the binding sites abolishes the reduction in reporter signal, confirming the MIR4435-2HG/miR-125a-5p/TAZ ceRNA axis [83]. Functionally, overexpression of MIR4435-2HG or TAZ increases levels and the expression of downstream Wnt pathway genes (β-catenin, c-Myc, cyclin D1), enhances viability, colony formation, migration and invasion, and reduces apoptosis; in contrast, miR-125a-5p exerts the opposite effect, lowering TAZ and Wnt signaling and inhibiting proliferation and migration [83]. Taken together, these examples show that, even in the absence of explicit matrix stiffness manipulation, ceRNA networks centered on LOX, FAK, YAP1 and TAZ provide a ncRNA regulatory layer over key mechanotransduction hubs, with the potential to modulate how tumor cells perceive and respond to a mechanically altered microenvironment (Figure 4).

Competing endogenous ceRNA networks describe post-transcriptional regulatory interactions in which different RNA species (lncRNAs, circRNAs, mRNAs and pseudogene-derived transcripts) share miRNA response elements (MREs) and can modulate each other’s expression by competing for a common pool of microRNAs. This framework provides a mechanistic basis to connect stiffness-driven changes in ncRNA abundance with downstream control of mechanotransduction hubs, while remaining highly context- and abundance-dependent (see Section 3) [81].

## 3. Future Directions and Perspectives

Key gaps include: (i) systematically validating mechanosensitive ncRNAs using controlled stiffness models (tunable hydrogels/organotypic matrices) with clear ‘soft vs. stiff’ definitions; (ii) directly quantifying ECM mechanics after ncRNA gain/loss-of-function using complementary biophysical readouts (e.g., atomic force microscopy (AFM) indentation, bulk rheology, traction force microscopy); (iii) separating tumor-cell versus stromal/immune-cell ncRNA programs, including extracellular vesicle cargo, under matched mechanical conditions; and (iv) quantifying transcript stoichiometry and localization to establish when ceRNA competition is plausible [82,95,96,97,98,99].

From a translational perspective, mechanosensitive ncRNAs could be leveraged as (a) therapeutic targets (e.g., antisense oligonucleotides, siRNA, CRISPRi/CRISPRa) and/or (b) biomarkers reflecting mechanical remodeling, including circulating or exosomal ncRNAs. However, delivery and target engagement can be hindered by dense, highly crosslinked ECM and elevated interstitial pressure in rigid tumors. Rational combinations that normalize or ‘soften’ the microenvironment (e.g., anti-fibrotic strategies, LOX/FAK/ROCK-axis modulation) may improve penetration and efficacy of ncRNA-directed therapeutics and should be prioritized in preclinical models that capture stiffness-dependent tumor–stroma feedback loops [82,95,96,97].

An additional priority is to expand mechanosensitive ncRNA frameworks beyond tumor-cell intrinsic regulation. Stiffness-driven activation of stromal populations (e.g., cancer-associated fibroblasts, endothelial cells and immune cells) can reprogram ncRNA expression and, in turn, alter paracrine signaling and extracellular vesicle cargoes that reinforce tumor progression. Integrating single-cell and spatial transcriptomics with stiffness mapping (e.g., elastography or AFM-based measurements) could clarify which ncRNA programs are initiated in stromal versus malignant compartments and how these programs propagate across the tumor microenvironment.

From a therapeutic standpoint, mechanosensitive ncRNAs may serve as actionable nodes for intervention or as biomarkers to stratify patients with highly fibrotic/stiff tumors. However, targeting stiffness-regulated ncRNAs (e.g., with antagomiRs, mimics or antisense oligonucleotides) will likely require addressing delivery barriers imposed by dense ECM and elevated interstitial pressure. Rational combinations that concurrently modulate ECM mechanics (e.g., anti-fibrotic/anti-crosslinking strategies) and ncRNA activity may improve efficacy and reduce adaptive resistance [82,95,96,97].

Finally, ceRNA network models should be interpreted cautiously in the context of mechanobiology. Quantitative studies indicate that effective miRNA ‘sponge’ activity depends strongly on stoichiometry (relative abundance of miRNAs, targets and competing transcripts), the number/affinity of miRNA response elements, and subcellular localization—factors that can vary substantially between tumor and stromal cells and across stiffness states. Accordingly, putative stiffness-linked ceRNA circuits should be supported by quantitative measurements of RNA copy numbers, binding site occupancy and mechanosensitive kinetics [98,99,100].

## 4. Conclusions

The evidence reviewed here indicates that extracellular matrix stiffness is not a passive property of the tumor microenvironment, but an active regulator of gene expression acting through defined mechanotransduction pathways. Changes in tissue Young’s modulus driven by collagen crosslinking, CAF-mediated remodeling or fibrosis activate integrins, focal adhesions and cytoskeletal reorganization, converging on mechanosensitive hubs such as LOX, FAK, YAP1 and TAZ. Together, these nodes form an ECM–adhesions–nucleus axis that sustains proliferative, invasive, stem-like and drug-resistant phenotypes and participates in pre-metastatic niche formation. Within this framework, mechanosensitive microRNAs and lncRNAs emerge as a critical post-transcriptional layer. Across models with tunable stiffness hydrogels in mammary, oral, hepatic and pulmonary systems, substrate rigidity profoundly reprograms intra- and extracellular miRNA and lncRNA profiles, shifting the balance between oncogenic and tumor-suppressive networks. Rather than acting as generic markers of stiffness, stiffness-regulated miRNAs and lncRNAs converge on specific modules, including PI3K/Akt and MAPK signaling, EMT and invasion programs, metabolic rewiring, apoptosis/autophagy and angiogenesis. In parallel, lncRNAs such as NEAT1, PVT1, SNHG8, satellite lncRNAs (HS2/HS3) or lnc-ZNF136 illustrate how these transcripts can function as both sensors and effectors of matrix stiffness in tumor and stromal cells. ceRNA circuits connecting ncRNAs with LOX, FAK, YAP1 and TAZ (for example, LINC01270–/HIF1A-AS2–miR-29c–LOX, KCNQ1OT1–miR-211-5p–Ezrin/FAK, LINC01811–miR-214-3p–YAP1 and MIR4435-2HG–miR-125a-5p–TAZ) further show that ncRNAs fine-tune the intensity and consequences of classical mechanotransduction pathways.

## Figures and Tables

**Table 1:** Physiological versus pathophysiological stiffness ranges for tissue.

Tissue	Physiological Stiffness	Pathophysiological/Tumor Stiffness	Typical Modality	Ref.
Liver	<6 kPa (healthy)	≈8 kPa (F3), ≥12.5 kPa (F4/cirrhosis)	Transient elastography/shear wave elastography	[64]
Breast	≈0.2 kPa (normal)	>4 kPa (tumor; reported ranges vary)	AFM/elastography	[65]
Colorectal/colon	≈0.5–1 kPa (normal)	≈2–8 kPa (CRC; reported ranges vary)	AFM/indentation	[66]

**Table 2:** Stiffness-regulated miRNAs directly stiffness-validated.

miRNA	Model/Cell Type	Mechanical Context	Key Target(s)/Hub	Reported Effect	Evidence	Ref.
miR-1246 (↑)	OSCC tumor cells	0.3 kPa and 0.9 kPa for our soft and stiff hydrogels	miRNome shift (multiple targets)	Associated with EMT-related and invasive phenotypes under stiffness	Direct	[70]
miR-17-5p (↑)	HCC tumor cells	Stiff ~16 kPa vs. soft ~6 kPa	PTEN/PI3K–AKT	Promotes proliferation and survival in stiff matrices	Direct	[71]
let-7d-5p (exosomal ↑)	HCC tumor stroma communication	Stiff ~16 kPa vs. soft ~6 kPa	HMGA2	Supports pro-tumor microenvironment remodeling	Direct	[72]
miR-365a-5p (exosomal ↑)	HCC tumor stroma communication	Stiff ~16 kPa vs. soft ~6 kPa	TRPC4AP/actin dynamics	Enhances actin remodeling and motility-associated programs	Direct	[72]
miR-3682-3p (↑)	HCC tumor cells	Stiff ~25.6 kPa vs. 0.4 Soft	PHLDA1	Increases growth and invasion on stiff matrices	Direct	[73]
miR-200c-3p (↓); miR-125a-5p (↓)	HCC tumor cells	Stiff ~16 kPa vs. soft ~6 kPa	N/A	Downregulated in metastatic patient	Direct	[70]

↑ High expression, ↓ Low expression.

**Table 3:** Summary of stiffness-regulated lncRNAs and key targets/hubs, cell-type context, and evidence type.

lncRNA	Model/Cell Type	Mechanical Context	Axis/Key Hub	Reported Effect	Evidence	Ref.
NEAT1 (lncRNA)	U2OS, 143B, and MDA-MB-231	soft (3 kPa) versus stiffer (40 kPa)	Mechanotransduction (YAP/TAZ-related programs)	Increased paraspeckle number	Direct	[76]
lnc-ZNF136 (lncRNA)	Ovarian cancer cells	1 kPa (soft), vs. 60 kPa (stiff)	PLEC and TNS2	Promotes focal adhesions	Direct	[11]
HS2/HS3 (lncRNAs)	CAFs (iCAFs)	Stiff ~50 kPa vs. soft ~0.2 kPa	Inflammatory CAF gene programs	Stiffness-induced CAF activation and cytokine signaling	Direct	[78]
LINC00894 (lncRNA)	Ovarian cancer tumor cells	Stiff PDMS ~106 kPa vs. ~25 kPa	Mechanosensitive transcriptional programs	Supports proliferation/migration in rigid environments	Direct	[7]
SNHG8 (lncRNA)	Ovarian cancer tumor cells	Stiff PDMS ~106 kPa vs. ~25 kPa	Mechanosensitive transcriptional programs	Decreased the efficiency of homologous recombination	Direct	[7]
PVT1 (lncRNA)	Ovarian cancer tumor cells	0.5 kPa (soft), vs. 8 kPa (stiff)	PVT1/YAP–TAZ axis	Tumor progression and chemoresistance	Direct	[77]
SNHG9 (lncRNA)	Breast tumor cells	Stiffness-associated	LATS1	Oncogenic signaling related to proliferation, survival and migration	Indirect	[80]
MIR4435-2HG (lncRNA)	Neuroglioma	Stiffness association inferred	MIR4435-2HG/miR-125a-5p/TAZ	Enhances viability, colony formation, migration and invasion	Indirect	[83]

## Data Availability

No new data were created or analyzed in this study.

## Abbreviations

The following abbreviations are used in this manuscript:

ECM	Extracellular matrix
TME	Tumor microenvironment
ncRNA	Non-coding RNA
miRNA	MicroRNA
lncRNA	Long non-coding RNA
circRNA	Circular RNA
ceRNA	Competing endogenous RNA
ECM stiffness	Extracellular matrix stiffness
AFM	Atomic force microscopy
kPa	Kilopascal(s)
LOX	Lysyl oxidase
FAK	Focal adhesion kinase
YAP1	Yes-associated protein 1
TAZ	Transcriptional co-activator with PDZ-binding motif (WWTR1)
CAF(s)	Cancer-associated fibroblast(s)
iCAF	Inflammatory cancer-associated fibroblast
CSC(s)	Cancer stem cell(s)
EMT	Epithelial–mesenchymal transition
MMP(s)	Matrix metalloproteinase(s)
HA	Hyaluronic acid
RTK(s)	Receptor tyrosine kinase(s)
LINC complex	Linker of nucleoskeleton and cytoskeleton complex
HCC	Hepatocellular carcinoma
OSCC	Oral squamous cell carcinoma
TNBC	Triple-negative breast cancer
HUVEC(s)	Human umbilical vein endothelial cell(s)
SASP	Senescence-associated secretory phenotype
